# A Text Messaging Intervention (Txt4HappyKids) to Promote Fruit and Vegetable Intake Among Families With Young Children: Pilot Study

**DOI:** 10.2196/formative.8544

**Published:** 2018-07-06

**Authors:** Julianne Mary Power, Andrea Bersamin

**Affiliations:** ^1^ Center for Alaska Native Health Research Institute of Arctic Biology University of Alaska Fairbanks Fairbanks, AK United States

**Keywords:** fruits and vegetables, nutrition education, nutrition intervention, young children, text messaging

## Abstract

**Background:**

Increasing fruit and vegetable intake among low-income populations, especially children, is a priority for United States federal food assistance programs. With over 49 million federal food assistance program recipients, cost-effective and efficient methods are needed to effectively deliver nutrition education to such a large population.

**Objective:**

The objective of our study was to examine the preliminary efficacy and acceptability of a text messaging intervention, Txt4HappyKids, to promote fruit and vegetable intake among families with young children.

**Methods:**

The intervention was evaluated using a pre-post study design. Parents (N=72) in Alaska were recruited from venues that serve a predominantly low-income population to participate in an 11-week intervention based on social cognitive theory. Parents received two texts per week promoting child fruit and vegetable intake. Behaviors, self-efficacy, and attitudes related to fruit and vegetable intake were measured at baseline and postintervention. Perceived changes in behaviors and open-ended feedback were also collected postintervention.

**Results:**

Of all participants, 67.3% (72/107) completed the intervention. We found no changes in behavior (*P*=.26), self-efficacy (*P*=.43), or attitudes (*P*=.35) related to fruit and vegetable intake from pre- to postintervention. Completers reported that since their participation in Txt4HappyKids, 92% (66/72) served more fruits and vegetables to their child because they thought fruits and vegetables were beneficial, 86% (62/72) tried to follow a healthier diet, 85% (61/72) tried different ways of preparing fruits and vegetables, and 81% (58/72) were more aware of the foods their child consumes. Additionally, 79% (57/72) of completers thought that Txt4HappyKids was credible, 71% (51/72) found texts useful, and 82% (59/72) would recommend it to a friend.

**Conclusions:**

A text messaging intervention was not sufficient to increase fruit and vegetable intake among families with young children. However, parents felt positively impacted by Txt4HappyKids and were receptive to nutrition information, despite the absence of face-to-face contact. High satisfaction among completers indicates that text messaging may be an acceptable complement to budget-constrained nutrition programs. These findings are an important first step in developing larger multi-level interventions utilizing mobile technology; however, a more rigorous evaluation of the Txt4HappyKids intervention is warranted.

## Introduction

In the United States 39.8% of adults and 18.5% of youth are obese, increasing the likelihood that they will develop cardiovascular risk factors that can lead to chronic diseases [1]. Fruit and vegetable (FV) consumption can protect against obesity; however, less than 18% of adults and 10% of youth meet the recommendations for FV intake [2-4]. Low-income populations are at an even greater risk for poor dietary patterns and are disproportionately impacted by obesity [4,5]. Understanding effective strategies to increase FV consumption among low-income people, low-income children in particular, could help to reduce diet- and obesity-related health disparities in this population.

Increasing FV consumption among low-income populations, especially children, is a priority for US federal food assistance programs such as the Supplemental Nutrition Assistance Program (SNAP) and the Women, Infants, and Children Program (WIC). Most interventions to increase FV intake among children involve nutrition education that is delivered in person through primary care, home visits, or school-based programs [6,7]. Although this strategy has resulted in small, significant increases in FV consumption at the individual level, face-to-face approaches are generally resource intensive and cannot be implemented at the population level. More cost-effective and efficient methods are needed to effectively deliver nutrition education to over 42 million SNAP [8] and 7 million WIC [9] participants in the United States.

Texting is an ideal tool to promote healthy behaviors among hard-to-reach populations, such as participants in US federal food assistance programs, for a number of reasons. First, the technology is ubiquitous. More than 90% of adults in the United States own a cell phone and more than 80% of cell phone owners report sending or receiving text messages via their phone [10,11]. Cell phone ownership is prevalent across income groups, and approximately 85% of adults with an annual income below $30K own a cell phone [11]. Second, text messaging is personal, and texts are important to recipients. Approximately 90% of text messages are read within 3 minutes [12]. Finally, text messaging is a cost-effective way to distribute health information on a large scale, thus reducing health service costs and participant burden [13] and improving reach to traditionally underserved populations [14].

Federal food assistance programs in the United States, such as SNAP and WIC, are shifting toward public health approaches for obesity prevention [15] and a growing number are incorporating text messaging into their programming [16,17]. Although results from these programs have not yet been published, studies demonstrate that text messaging can effectively promote diabetes and weight management, medication compliance, smoking cessation, and other health behaviors [18-25]. Although most of these studies have examined the use of text messaging to enhance treatment outcomes in clinical settings, less information is available on how text messaging can be used for preventive behaviors such as FV consumption [26-28].

A text messaging intervention to promote FV intake may be a convenient and cost-effective way for low-income parents to receive health-related information about their children. This paper reports on the preliminary efficacy and acceptability of a text messaging intervention to promote FV consumption among parents of young children from low-income families in Fairbanks, Alaska. Alaska was an ideal place to pilot test this program because insufficient FV intake is a common dietary shortcoming resulting from unique environmental factors. Additionally, low population density across the state and lack of affordable travel between communities significantly limits traditional, face-to-face nutrition education [29].

## Methods

### Study Design

Txt4HappyKids is an 11-week, theory-based intervention that sends parents twice weekly text messages encouraging them to serve more FV to their child. The intervention was evaluated using a pre-post study design. Parents completed a self-administered questionnaire to assess behaviors, self-efficacy, and attitudes related to FV intake at baseline and postintervention.

Participants were recruited using convenience sampling at the following venues that serve a predominantly low-income population: Head Start (n=18); WIC (n=18); the public library (n=12); and a free family health fair (n=59) in Fairbanks, Alaska. Fairbanks is the second largest city in Alaska with a population of approximately 30,000 people. Inclusion criteria were being the parent or guardian of a young child (no age specified) and having an unlimited texting plan on a mobile phone. The unlimited texting plan was a necessary inclusion criterion to ensure that participants would not incur charges from intervention-related text messages, which this pilot study could not reimburse.

Researchers set up an information table at each location to recruit parents in person to participate in the study. Researchers collected informed consent and administered the baseline questionnaire to interested parents. Parents then provided their email and cell phone number to complete study enrollment. Participation was incentivized so that parents received a small prize, such as a water bottle, upon enrollment. Owing to logistical constraints, the postintervention questionnaire was administered online via an email link. Follow-up was incentivized by offering participants a $25 gift card to a local grocery store upon completion of the follow-up assessment. All procedures were approved by the University of Alaska Fairbanks Institutional Review Board for human subjects.

#### Intervention

Text message development was guided by the social cognitive theory (SCT) and messages were designed to address the personal, behavioral, and environmental factors related to FV intake [30] (Table 1). Messages were limited to 160 characters and content was adapted from the TXT4Tots library of evidence-based messages created by the US Department of Health and Human Services and the American Academy of Pediatrics [31].

**Table 1:** Development of Txt4HappyKids using the social cognitive theory. Social cognitive theory factors appear in bold and predictive factors are nested.

Factors			Explanation of predictive factors		Example text
**Personal**					
	Knowledge	Provide information about the health benefits of consuming fruits and vegetables		Eating fruits & veggies helps your child build strong muscles and bones. Give your child the gift of health by serving fruits & veggies with every meal.	
	Preference	Portray fruits and vegetables as tasting good and something children enjoy eating		Apples are on sale for 1.49/lb @ Fred Meyer! Peel, core & chop. Add water & ground cinnamon. Cook for 30 min until soft, then mash. Kids love warm applesauce!	
	Time	Portray shopping and cooking with children as a great way to spend quality time together		Kids love to be helpful! Let them help with dinner by washing the fruits & veggies, stirring, or measuring. This is a great way to spend quality time together!	
**Behavioral**					
	Self-efficacy	Improve skills related to preparing fruits and vegetables by providing recipes and other tips		Frozen broccoli has as much fiber as fresh broccoli! Microwave until tender & toss with some olive oil, lemon juice, garlic powder, salt & pepper!	
**Environmental**					
	Cost	Announce sales at grocery stores so more fruits and vegetables are available at home		Satsuma Mandarins are on sale @ Fred Meyer for $5.99/5 lb box! Keep your kids on the fast track to health with this sweet snack that is quick & easy to eat.	
	Role models	Motivate parents to be positive role models for their children by consuming fruits and vegetables		Your kids look up to you! Set another good example for your kids by eating fruits & veggies with your meals & as snacks.	

The research team developed 61 text messages that were pilot tested with 15 low-income women through individual or group interviews. Individual interviews (n=3) took place in the waiting room at the Fairbanks WIC clinic. The group interview (n=12) took place during a class at a local organization. During these interviews, women were provided with a list of text messages and participated in an informal discussion about message preferences, such as which messages they liked, disliked, and why. Notes from these interviews were compiled into a spreadsheet and messages were ranked based on women’s preferences. Messages were then revised accordingly and selected for use in the Txt4HappyKids intervention. Text messages were delivered using an online text marketing service.

#### Instrument

The baseline questionnaire was administered in person and the postintervention questionnaire was administered electronically via SurveyMonkey. Scale items were adapted from the Food Stamp Program Fruit and Vegetable Checklist [32] and the Fruit and Vegetable Inventory [33], which were developed at the University of California Davis and validated for use in a low-income population [34-36].

### Variables Measured

Behaviors were measured using a single scale comprising the following four items related to serving FV to children: how often (1) participants serve meals with FV, (2) their child eats FV as a snack, (3) their child eats more than one kind of vegetable a day, and (4) their child eats more than one kind of fruit a day. Response options ranged from 1 (rarely) to 5 (always); therefore, higher scores represented more frequent behaviors that involved serving FV to children (alpha=.84, *r̅*=.56).

Self-efficacy was measured using a single scale consisting of six items related to shopping for and serving FV to children. Participants were asked how strongly they agreed with the following statements: I feel that I can (1) serve more FV as a snack, (2) buy more vegetables next time I shop, (3) serve meals or snacks with more fruit during the next week, (4) serve two or more servings of vegetables at dinner, (5) serve meals with more vegetables during the next week, and (6) add extra vegetables to casseroles and stews. The response options ranged from 1 (strongly disagree) to 5 (strongly agree); therefore, higher scores represented greater self-efficacy related to buying and serving children more FV (alpha=.88, *r̅*=.54).

Attitudes were measured using a single scale consisting of five items related to perceived benefits of serving FV to children and role modeling FV intake. Participants rated their agreement with the following statement: I feel that (1) I am helping my child’s body by serving them more FV, (2) my child may develop health problems if they do not eat FV, (3) eating FV will help my child succeed in school, (4) I might be able to influence my child to be healthier by eating FV more often, and (5) I would set a good example for my child if I ate more FV. Response options ranged from 1 (strongly disagree) to 5 (strongly agree); therefore, higher scores represented more favorable attitudes toward serving FV and being a positive role model for FV intake (alpha=.88, *r̅*=.58). In addition to these measures, at postintervention, participants answered questions related to perceived changes in behaviors related to FV intake. These questions used the stem “Because of the information that you learned from Txt4HappyKids…” and included items such as “Have you tried different ways of preparing fruits and vegetables?” and “Have you tried to follow a healthier diet?” with response options of “No,” “Yes,” and “Don’t Know.” Participants also answered questions related to intervention satisfaction, such as “Would you recommend Txt4HappyKids to a friend?” Finally, participants answered open-ended questions, including “What did you like most about Txt4HappyKids?” and “What changes would make Txt4HappyKids better?”

### Analysis

Frequencies were calculated using the IBM SPSS Statistics 19 Software [37] to examine participant demographics and general response patterns. Fisher’s exact tests were used to examine the differences in the demographic characteristics between completers and noncompleters. A paired samples *t* test was used to compare paired pre- and postintervention responses to each scale. Open-ended questions were coded for concepts and themes by two separate coders using the constant comparative method of analysis in Microsoft Excel [38]. Coders discussed the disagreements in coding until a consensus was reached. Only participants who completed both the baseline and postintervention assessments were included in the final analysis.

## Results

### Participant Characteristics

Of all participants, 67.3% (72/107) completed the intervention. The demographic characteristics of completers (N=72) are presented in Table 2. The majority of completers were white females between 25 and 34 years of age with some college education and a child under 5 years old. Almost half of them reported receiving food assistance during the last 12 months. There were no differences between completers and noncompleters in terms of race (*P*=.07) or whether food assistance was received in the last 12 months (*P*=.53); however, males were significantly more likely to be noncompleters than completers (*P*=.01).

### Preliminary Efficacy

Estimates of behavior, self-efficacy, and attitudes related to FV intake were close to optimal at baseline. A paired samples *t* test showed that there were no significant changes in the participant responses to these measures postintervention (Table 3). However, 92% (66/72) of completers reported that since their participation in the Txt4HappyKids intervention, they served their child more FV because they thought FV were beneficial, 86% (62/72) tried to follow a healthier diet, 85% (61/72) tried different ways of preparing FV, and 81% (58/72) were more aware of the food their child consumes. Additionally, 83% (60/72) and 78% (56/72) of completers agreed or strongly agreed that more fruits and vegetables, respectively, were available in their home since their participation in the Txt4HappyKids intervention.

**Table 2:** Demographic characteristics of completers (N=72) in the Txt4HappyKids intervention.

Variable		n (%)
**Sex**		
	Female	70 (99)
**Race**		
	White	50 (69)
	Alaska Native	9 (12.5)
	Other	13 (18)
**Age**		
	Under 25 years	12 (17)
	25-34 years	37 (51)
	35-44 years	16 (22)
	45-54 years	7 (10)
**Education**		
	No college	14 (20)
	Some college	57 (80)
**Age of children^a^**		
	Under 5 years	52 (72)
	5-8 years	40 (56)
	9-17 years	24 (33)
**Income proxy**		
	Food assistance^b^	35 (49)

^a^Participants could report the age of more than one child in their household; therefore, response options were not mutually exclusive.

^b^Received food assistance in the last 12 months from SNAP or WIC; emergency food banks, food pantry, soup kitchen; or meals served at a food kitchen or community site.

**Table 3:** Changes in behavior, self-efficacy, and attitudes related to fruit and vegetable intake using a paired samples *t* test (N=72).

Measure	Preintervention, mean (SD)	Postintervention, mean (SD)	Mean difference, (SD)	*P* value
Behavior^a^	3.51 (0.92)	3.61 (0.80)	0.10 (0.73)	.26
Self-efficacy^b^	3.97 (0.62)	4.04 (0.59)	0.06 (0.69)	.43
Attitudes^b^	4.47 (0.55)	4.54 (0.52)	0.06 (0.57)	.35

^a^Response options ranged from 1 (rarely) to 5 (always).

^b^Response options ranged from 1 (strongly disagree) to 5 (strongly agree).

### Program Acceptability

Overall, parents liked the intervention; 79% (57/72) of completers thought Txt4HappyKids was very credible and 71% (51/72) found the text messages very useful. In addition, 67% (48/72) of completers reported wanting to receive more texts about consuming FV and 82% (59/72) stated that they would recommend the program to a friend; 76% (55/72) of completers felt that they received the right number of messages, and no one reported that they received too many messages.

### Responses to Open-Ended Questions

Parents were highly positive regarding their experience with the intervention; 89% (64/72) of completers responded to the question “What did you like most about Txt4HappyKids?” The most frequently mentioned components were the sales announcements (22/64 respondents) and the recipes (11/64 respondents). Respondents noted that sales announcements “helped me plan what to buy” and that recipes “helped me help my son explore new foods.” Of 64 respondents, 19 stated that the intervention gave them new ideas: “it wasn’t just ‘you should do this or that.’ It was actually ideas” and “I wouldn’t have thought about it (the ideas) without the text.” Of 64 respondents, 6 talked about the simplicity of the program, stating that it was “convenient, consistent and free” and “easy, short and to the point.” Of 64 respondents, 6 noted that the texts served as useful reminders, stating “I think one of the most helpful things is that it puts the idea in your mind and you think about it through the day” and “I also liked the reminder that if I eat better, my kids will see that as an example. It’s easy to forget that at times.” Another respondent said the program was “great encouragement for those who are trying to feed their kids healthier foods and a great reminder for those parents already feeding their kids healthy.”

Furthermore, 67% (48/72) of completers responded to the following question: “What changes would make Txt4HappyKids better?” Eighteen out of 48 respondents did not think that there should be any changes. Of 48 respondents, 8 (17%) wanted to receive texts more frequently, such as every other day, every time there was a sale on produce, or for more than 11 weeks; furthermore, 2 respondents (4%) wanted an interactive element and 2 respondents (4%) expressed interest in receiving emails or being able to access a website. Regarding message content, 7/48 respondents (14.5%) wanted more recipes, 3/48 respondents (6%) wanted more sales information, and 2/48 respondents (4%) suggested providing links to additional information. Furthermore, 3/48 respondents (6%) suggested including more tailored message content, such as “different levels of information,” “more ideas of how to get my kids to eat fruits and vegetables,” and “more infant friendly ideas.”

## Discussion

This pilot study examined the preliminary efficacy and acceptability of a text messaging intervention to promote FV intake among families with young children. We found no changes in behavior, self-efficacy, or attitudes related to FV intake from pre- to postintervention. However, the majority of parents felt positively impacted by the intervention and reported high satisfaction. This is important because parents were engaged and receptive to the nutrition information received, despite the absence of face-to-face interaction, which can be costly and resource intensive. A recent study by Pedersen et al showed greater increases in FV intake among adolescents with a higher engagement in a text message-based feedback intervention [27]. High levels of engagement demonstrate the acceptability of this low-cost intervention.

Parents expressed particular satisfaction with texts about sales and recipes and the reassurance of receiving reminders and encouragement to serve more FV. The most frequently used word to describe what parents liked most about the program was “ideas.” Parents felt that the program provided ideas for new ways to prepare and serve FV, which may indicate feelings of ownership of the information provided. These findings suggest improvements in the self-efficacy of parents. Self-efficacy is the most important construct in SCT because individuals with a greater sense of self-efficacy feel more capable of changing their behavior, despite barriers [39].

There was no change in pre-post measures, which may be explained in part by the high percentage of completers (92%) who reported at baseline that the foods their child consumed were somewhat or very healthy. Additionally, 51 out of 72 completers (71%) reported that they served meals with FV very often or always. In other words, given the favorable responses at baseline, there was little room for improvement. However, only 32 out of 72 (44%) and 23 out of 72 (32%) completers reported that they were already serving the recommended amounts of fruits and vegetables per day, indicating that there was still room to improve behaviors related to FV intake in this population. Parents may recognize that FV are an important part of a healthy diet but may not be aware of the daily recommended amount for children, which could negatively influence parental encouragement of child FV consumption [40]. Another explanation is that our instrument did not accurately measure child FV intake, which may have resulted in a ceiling effect and limited our ability to detect changes. Although scale items were adapted from measures that have been validated for a low-income population, items were rewritten to assess child, as opposed to parent, FV intake. High Cronbach alphas and moderate interitem correlations indicated an acceptable scale reliability. However, additional validity testing is needed. Using different modes to administer the questionnaire at pre- and postintervention may have introduced additional variance in the participant responses, which may also have contributed to the null findings.

It is also possible that the number of text messages sent or the intervention duration, or both, may have been insufficient to observe an improvement from pre- to postintervention. Although the use of text message-based interventions for changing health behaviors has increased in recent years [25], it is possible that text messaging is not sufficient as a stand-alone intervention to increase FV intake. Many other interventions utilizing text messaging have provided supplementary materials, including interactive websites [18], consultations or education sessions before or during the intervention [19], printed materials [20], and self-monitoring components [21]. Additionally, many text message-based interventions have been implemented in clinical settings so that participants received the standard of care plus text messaging [22-24]. Promising effects of such interventions are likely the synergistic effects of multiple components because these studies did not isolate the effects of text messaging on outcomes. Electronically delivered health interventions, however, have shown promise for effecting change in dietary behaviors, such as FV intake [28,41,42]. Future research is needed to explore whether such materials would strengthen program effects.

A more rigorous evaluation of the Txt4HappyKids intervention is needed. However, given our interest in understanding participant satisfaction, an important first step in developing larger multi-level interventions utilizing mobile technology, the pre-post study design was appropriate for this pilot study. One limitation of this study design was that these findings can only be interpreted in the context of our convenience sample, which may not represent the general population. Parents were recruited primarily from a family health fair; therefore, they may have been more interested in health and nutrition compared with the general population. Additionally, only results from completers are presented in this study, which may have yielded higher reported satisfaction with the intervention. Another limitation of this study was that the participants were relatively homogenous, and only half of them were considered as low income according to the proxy measure of whether food assistance was received during the last 12 months. However, it is possible that we underestimated the numbers of low-income participants because some participants likely met the eligibility requirements for food assistance but may not have applied for or received it. Other factors, such as child age (in the case of WIC), could have played a larger role in determining whether food assistance was received. Future research should target more diverse populations, which would provide important insights into cultural and community differences regarding how the program is perceived, thereby expanding the generalizability of the intervention.

The findings from the current research demonstrate that a text messaging intervention to promote FV intake did not change behaviors related to FV intake; however, text messages did create positive perceptions of behavior change among parents of young children. High levels of satisfaction with the intervention among completers indicates that text messaging may be an acceptable complement to federal food assistance programs, such as SNAP or WIC, which have limited funding to deliver nutrition education to millions of people. Using text messaging, time-constrained staff can more effectively reach a large number of clients. Additionally, incorporating text messaging into SNAP or WIC programming would support the federal priority to move toward public health approaches and may increase satisfaction with the existing nutrition education by providing information in a format that clients prefer [43,44].

## Abbreviations

FV: fruit and vegetable
SCT: Social Cognitive Theory
SNAP: Supplemental Nutrition Assistance Program
WIC: Women, Infants, and Children Program
